# Case Report: Exceptional longevity in turner syndrome

**DOI:** 10.3389/fragi.2026.1805342

**Published:** 2026-06-10

**Authors:** Mateus Vidigal de Castro, João Paulo Limongi França Guilherme, Mayana Zatz

**Affiliations:** Human Genome and Stem Cell Research Center, University of São Paulo, São Paulo, SP, Brazil

**Keywords:** aging, genetic variants, health, longevity, resilience, turner syndrome

## Abstract

Turner Syndrome (TS), caused by complete or partial monosomy of chromosome X, is associated with increased morbidity and reduced life expectancy, largely driven by cardiovascular, endocrine, and metabolic complications. Here, we report the case of a 75-year-old woman with a confirmed diagnosis of non-mosaic TS (45,X) who remains clinically stable and functionally independent, beyond the expected survival for this condition. Notably, her mother is currently 101 years old, indicating a familial background of exceptional longevity. To explore the potential contribution of inherited genetic factors to this favorable phenotype, whole-genome sequencing (WGS) was performed in both the TS patient and her centenarian mother aiming to identify variants potentially associated with healthy aging and longevity. Hematological and biochemical analyses were subsequently used to functionally support the genetic and clinical findings, revealing preserved metabolic, inflammatory, and hematological profiles for the patient age. Together, these data suggest that a favorable genetic background may partially counterbalance the risks traditionally associated with X chromosome monosomy, contributing to preserved functional status into older age.

## 1 Introduction

Turner Syndrome (TS) affects approximately 1 in 2,500 live-born females and is associated with short stature, gonadal dysgenesis, infertility, and increased risk of metabolic and cardiovascular diseases (Cui et al., 2018). Life expectancy in Turner syndrome is significantly reduced compared with the general female population, as demonstrated in large national and longitudinal cohort studies, with estimates suggesting a reduction of approximately 1 decade or more, largely driven by cardiovascular mortality (Stochholm et al., 2006; Fuchs et al., 2019). Consequently, individuals with TS reaching advanced ages are uncommon and remain underrepresented in the literature, limiting current understanding of the mechanisms associated with long-term survival.

Recently, Kaur and O’Sullivan (2024) described a 76-year-old woman from New Zealand with TS diagnosed only late in life, presenting with mosaicism (45,X/46,X,rY) and a history of osteoporosis, coeliac disease, hypertension, and multiple complications. Her diagnosis was established after a hip fracture (Kaur and O'Sullivan, 2024). In contrast, the present report describes a Brazilian woman with classic non-mosaic Turner syndrome (45,X), diagnosed early in life and treated with long-term hormone replacement therapy, who has reached 75 years of age with preserved functional independence and clinical stability. Unlike the previously reported case, this patient underwent early oophorectomy followed by decades of continuous estrogen therapy and remains independent in daily activities, without major physical limitations. Most importantly, her mother, currently aged 101 years, exhibits exceptional longevity. These observations suggest that potentially inherited protective genetic factors, early diagnosis and sustained clinical management, may modulate the TS clinical course and contribute to survival into advanced age.

## 2 Case description

We report the case of a 75-year-old woman with a confirmed diagnosis of Turner syndrome (TS) who has reached advanced age as compared to the expected life span for this condition, who preserved functional independence. Her height is 1.38 m and weight: 40 kg. Despite multiple comorbidities, she remains clinically stable and independent in daily activities, with no significant physical limitations. She is the only child of a centenarian mother (currently 101 years old) while her father lived until 87 years of age, suggesting a familial genetic background potentially enriched for factors associated with healthy aging and longevity.

### 2.1 Diagnosis

The patient was initially diagnosed during adolescence through conventional karyotype analysis of peripheral blood cells. Cytogenetic testing was repeated at age 60, with analysis of 40 metaphase blood cells, all demonstrating monosomy X (45,X), with no evidence of mosaicism. The karyotype was further supported by whole-genome sequencing (WGS) performed on genomic DNA extracted from the patient’s peripheral blood. Median sequencing depth was calculated separately for the autosomes, chromosome X, and the SRY locus (chromosome Y) using the DepthOfCoverage tool from GATK v3.7. Non-Turner female samples showed mean chromosome X coverage comparable to the average coverage of autosomes, whereas the Turner syndrome sample exhibited mean chromosome X coverage approximately half that of the autosomes—similar to the pattern observed in males (defined as samples with mean SRY coverage >1×) (Table 1).

**TABLE 1:** Whole-genome sequencing coverage across autosomes, chromosome X, and the SRY locus.

IDs	Total reads	Autosomes coverage	Chr X coverage	SRY coverage
TS patient	116489223184	38.28	19.76	0
TS’ mother	85467279190	28.08	27.71	0
Older female 1	119399612582	39.23	38.38	0
Older female 2	87797159121	28.85	28.02	0
Older male 1	97793028402	32.13	16.57	6.98
Older male 2	114852017870	37.74	19.86	16.87

To further evaluate the possibility of mosaicism, we examined the VCF file generated from the same WGS dataset. Among the 109,292 variants located in non-pseudoautosomal regions of chromosome X, only 3,095 were called as heterozygous, while the vast majority were homozygous and showed balanced allele representation. This pattern is consistent with the presence of a single X chromosome and indicates that the deletion of one X chromosome is most likely complete, supporting a non-mosaic 45, X karyotype. In addition, WGS of both the TS patient and her centenarian mother (detailed methodology provided in the Supplementary Material) was used for exploratory analysis of genetic variants that have been consistently reported in population-based studies to be associated with traits related to healthy aging and extended lifespan.

### 2.2 Gynecological, hormonal, and surgical history

Bilateral oophorectomy at age 15, with immediate initiation of hormone replacement therapy (HRT) using estrogen and progesterone; At age 48, the patient underwent hysterectomy, and progesterone was discontinued, continuing estrogen-only HRT thereafter; About 4 years ago, she replaced the estrogen formulation and continues on daily estrogen until now. She has never received growth hormone (GH) therapy at any point in her life.

### 2.3 Clinical history and comorbidities

Hypertension (>20 years, treated with enalapril 5 mg/day), hypothyroidism (>15 years, on levothyroxine 37 μg/day), dyslipidemia (treated with pitavastatin 4 mg/day), horseshoe kidney with prior nephrolithiasis, bilateral hearing loss since age 54 (∼50% impairment), and osteoporosis without documented fractures. She also has a history of ischemic stroke without residual motor deficits, currently on antithrombotic therapy, and prediabetes. Additional conditions include urinary incontinence since age 44, two episodes of pneumonia of unknown etiology, cataracts treated surgically at age 50, and attention-deficit disorder.

### 2.4 Audiological assessment

Pure-tone audiometry performed at age 75 revealed bilateral sensorineural hearing loss, predominantly affecting mid-to high-frequency ranges. Speech reception thresholds (SRT) were 65 dB in the right ear and 60 dB in the left ear. Speech recognition scores remained relatively preserved at high intensity levels (100 dB), with word recognition of 84%–88% in the right ear and 88%–92% in the left ear. Overall, the findings are consistent with moderate bilateral hearing loss with partial preservation of speech discrimination.

### 2.5 Bone mineral density assessment

Dual-energy X-ray absorptiometry (DXA) performed at age 75 demonstrated reduced bone mineral density across multiple skeletal sites. The lumbar spine (L1–L4) showed a BMD of 0.920 g/cm^2^ (T-score: −2.2), consistent with osteopenia. The right femoral neck and total femur presented BMD values of 0.798 g/cm^2^ (T-score: −1.7) and 0.775 g/cm^2^ (T-score: −1.8), respectively, also within the osteopenic range. Notably, the distal radius (33%) exhibited a markedly reduced BMD of 0.570 g/cm^2^ (T-score: −3.5), meeting criteria for osteoporosis. Overall, these findings support a diagnosis of osteoporosis, predominantly driven by cortical bone loss at the forearm.

### 2.6 Cardiovascular follow-up

Given the increased cardiovascular risk associated with Turner syndrome, the patient undergoes regular cardiology follow-up approximately once per year. The most recent transthoracic echocardiogram (Supplementary Figure S1), performed at age 74, showed normal aortic root dimensions (2.3 cm), normal left atrial size, preserved ventricular dimensions, and normal left ventricular systolic function with an ejection fraction of 60%, with no evidence of aortic dilation or other structural cardiac abnormalities.

## 3 Results

### 3.1 Whole-genome analysis of variants associated with healthy aging and longevity

Based on the sum of known longevity alleles, the TS patient was shown to possess 60 (out of 120) favorable alleles, while her centenarian mother carries 55. When expressed on a scale from 0 to 100 (representing least to most favorable), the TS patient achieved a score of 50, whereas her centenarian mother obtained a score of 45.8. Importantly, this polygenic score was not intended to capture Turner syndrome–related risk, but to explore whether the patient harbors a genetic background enriched for variants associated with healthy aging and longevity. Although these polygenic profiles fall within an intermediate range (50% and 46%, respectively), both individuals carry a relevant number of variants previously associated with longevity. These findings support the hypothesis that the TS patient may possess a protective polygenic background that contributes to preserved health status and extended survival, even in the context of complete X monosomy and classical Turner syndrome traits. A complete list of the genetic variants included in this analysis is provided in the (Supplementary Table S1). Here, we highlight selected variants observed in homozygosity with potential biological relevance, aiming to explore mechanisms that may contribute to a protective phenotype (Table 2). Homozygosity may increase the likelihood that the functional effects of individual alleles are expressed. Importantly, no single variant is expected to determine clinical outcome; rather, their combined presence may contribute to a more favorable biological background.

**TABLE 2:** Longevity-associated genetic variants identified in the turner syndrome patient and her centenarian mother.

Mapped gene	rsID	TS patient	Mother	Favorable allele	Local frequency (%)*	Global frequency (%)*
Shared homozygous longevity-associated variants						
ABO	rs977371848	C/C	C/C	C	81.6	82.3
APOC1	rs4420638	A/A	A/A	A	84.6	82.8
APOE	rs429358	T/T	T/T	T	87.0	84.9
ASXL2	rs934073	C/C	C/C	C	70.8	68.1
CAMK4	rs10491334	C/C	C/C	C	81.3	83.2
DEFB136	rs9657521	A/A	A/A	A	69.7	70.5
IL6	rs2069837	A/A	A/A	A	90.1	91.8
LPA	rs10455872	A/A	A/A	A	96.2	94.0
LPL	rs268	A/A	A/A	A	99.3	98.4
PABPC4	rs3768321	G/G	G/G	G	85.8	85.1
TOMM40	rs2075650	A/A	A/A	A	89.6	86.6
ZW10	rs61905747	A/A	A/A	A	85.9	83.3
Differential zygosity longevity-associated variants						
LTA; TNF	rs1800629	G/G	G/A	G	89.3	84.4
KL	rs9536314	G/G	T/G	G	14.4	14.7
KL	rs9527025	C/C	G/C	C	14.4	15.7
CETP	rs7499892	C/C	C/T	C	78.3	80.9
ACE	rs4343	G/G	G/A	G	48.4	49.5

* The local frequency of the favorable allele was calculated based on data available in ABraOM (http://abraom.ib.usp.br), while the global frequency (across all populations) was calculated using data from IGSR (https://www.ensembl.org/index.html). Variants in the *KL* gene may be inherited within the same haplotype and were considered as a single value in the polygenic score.

Several longevity-associated variants were observed in homozygosity in both the TS patient and her centenarian mother, supporting the presence of a shared familial genetic background associated with healthy aging and extended survival. These include variants in genes related to lipid metabolism, such as *APOE* rs429358 (T/T), *APOC1* rs4420638 (A/A), *LPL* rs268 (A/A), and *LPA* rs10455872 (A/A); immune and inflammatory regulation, including *IL6* rs2069837 (A/A) and *DEFB136* rs9657521 (A/A); as well as genes implicated in biological processes relevant to healthspan and lifespan regulation, such as *ASXL2* rs934073 (C/C), *CAMK4* rs10491334 (C/C), and *ZW10* rs61905747 (A/A).

Furthermore, a distinct subset of longevity-associated variants present in heterozygosity in her centenarian mother were identified in homozygosity in the TS patient reinforcing their genetic background. For example, variants mapped to genes involved in cardiometabolic regulation and inflammatory control, key signaling pathways for healthspan. This pattern is consistent with a potential allele-dosage effect, whereby the homozygous state in the patient may enhance the cumulative impact of variants individually associated with healthy aging and longevity. Notably, the TS patient is homozygous for the *KL*-VS haplotype, defined by the rs9536314 (F352V) and rs9527025 (C370S) variants (G/G and C/C genotypes, respectively) in the *KL* (Klotho) gene.

The TS patient is also homozygous for the CETP rs7499892 variant (C/C), which is a major determinant of interindividual differences in lipid traits, particularly HDL cholesterol, a recognized marker of cardiovascular protection. Consistent with this genetic background, biochemical analysis revealed an HDL cholesterol level of 63 mg/dL, considered favorable in terms of cardiometabolic risk. Additionally, the patient is homozygous for the *LTA*/*TNF* rs1800629 (G/G), a genotype associated with lower transcriptional activity of the TNF promoter. In line with this genetic background, circulating TNF-α levels in plasma samples (limit of detection: 67.3 fg/mL) were below the assay’s detection threshold when evaluated using a Cytometric Bead Array (CBA) assay. While this finding is consistent with a low basal inflammatory profile, it should be interpreted with caution given the sensitivity limitations of the assay. Although this observation cannot be attributed to a single genetic variant, it is consistent with a phenotype characterized by attenuated chronic inflammation and preserved immune homeostasis.

In addition to TNF, we also measured two other major pro-inflammatory cytokines, IL-6 (limit of detection: 68.4 fg/mL) and IL-1β (limit of detection: 48.4 fg/mL), using the same method. The results showed that IL-6 was detectable (1585.67 fg/mL) and its level was within the expected range for healthy elderly women, consistent with values reported in aging populations without acute inflammatory conditions. IL-1β was not detected, remaining below the sensitivity threshold of the assay. Taken together, these findings further support the interpretation of a low systemic inflammatory profile in this individual. For the CBA assays, plasma was isolated from peripheral blood (collected in EDTA tubes) and promptly processed by centrifugation under standardized conditions. Plasma samples were analyzed in technical triplicates (10 μL per analyte). Serum was also obtained by centrifugation of peripheral blood collected in serology tubes with separating gel after 30 min of clot formation and subsequently used for the following biochemical analyses.

### 3.2 Hematological profile and serum biochemistry panel

As an assessment of health status, complete blood count and serum biochemical analyses were performed for the TS patient. Results were interpreted descriptively against established age-specific reference intervals. A healthy, age-matched 46,XX woman was included as an internal control solely to ensure analytical consistency (e.g., sample handling, instrumentation, and assay performance), rather than for biological or statistical comparison. Both samples were processed using the same equipment and under identical conditions, including non-fasting sample collection, reagent batch, and analytical protocols.

The hematological profiling of the TS patient revealed a stable complete blood count within the expected range for her age (Table 3). Minor findings included a slight increase in the percentage of mid-sized white blood cells and mild hypochromia. Overall, these findings indicate preserved hematological stability and adequate physiological adaptation, despite the diagnosis of Turner syndrome and the presence of multiple comorbidities.

**TABLE 3:** Hematological Parameters of TS patient.

Parameter group	Item	TS patient	Control	References range
White series	White blood cells	5.47 × 10^9^/L	5.26 × 10^9^/L	4.0–10.0
	Lymphocyte count	1.40 × 10^9^/L	1.94 × 10^9^/L	0.8–4.0
	Mid-sized cell count	0.54 × 10^9^/L	0.48 × 10^9^/L	0.0–0.8
	Granulocyte count	3.53 × 10^9^/L	2.84 × 10^9^/L	1.8–6.3
	Lymphocyte percentage	25.6%	36.8%	20–40
	Mid-sized cell percentage	↑ 9.8%	↑ 9.2%	0–8
	Granulocyte percentage	64.6%	54%	50–70
Red series	Red blood cells	4.67 × 10^9^/L	4.52 × 10^9^/L	3.5–5.5
	Hemoglobin	12.9 g/dL	12.1 g/dL	11–16
	Hematocrit	41.6%	40.2%	37–49
	Mean corpuscular volume (MCV)	88.9 fL	89.1 fL	80–100
	Mean corpuscular hemoglobin (MCH)	27.6 pg	↓ 26.7 pg	27–34
	Mean corpuscular hemoglobin concentration (MCHC)	↓ 30.9 g/dL	↓ 30.0 g/dL	32–36
	Red cell distribution width – CV (RDW-CV)	11.8%	11.8%	0–16
	Red cell distribution width – SD (RDW-SD)	41.3 fL	52.2 fL	37–55
Platelet Parameters	Platelet count	258 × 10^9^/L	247 × 10^9^/L	100–300
	Mean platelet volume (MPV)	10.8 fL	11.3 fL	7–12
	Platelet distribution width (PDW)	13.2%	12.3%	8–18
	Plateletcrit (PCT)	0.279	0.279	0.1–0.282
	Platelet large cell ratio (P-LCR)	29.6%	35.6%	13–43
	Large platelet count (P-LCC)	76 × 10^9^/L	88 × 10^9^/L	10–100

Serum biochemical profiling of the TS patient (Table 4) indicates an overall preserved metabolic status. Although total cholesterol and LDL cholesterol levels were mildly elevated, the lipid profile remains favorable for age, particularly due to high HDL cholesterol (63 mg/dL), a well-recognized cardioprotective marker. Triglyceride levels were within the normal range, further supporting a low atherogenic risk profile. Markers of hepatic, renal, muscular, and protein metabolism were largely within reference ranges for age, indicating preserved systemic homeostasis despite the presence of lifelong monosomy X and age-related comorbidities. Iron metabolism parameters revealed reduced ferritin levels without anemia, a common finding in elderly women, while calcium levels were within normal limits. Zinc levels were mildly elevated, likely reflecting individual variation or supplementation, and were not associated with clinically relevant abnormalities.

**TABLE 4:** Serum Biochemistry panel of TS patient.

Function	Item	TS patient	Control	References range*
*Lipid profile*	Total cholesterol	220 mg∕dL (↑)	260 mg∕dL (↑)	<200 mg∕dL
	HDL cholesterol	63 mg∕dL	81 mg∕dL	>40 mg∕dL
	Non-HDL cholesterol	157 mg∕dL	179 mg∕dL (↑)	<160 mg∕dL
	LDL cholesterol	128 mg∕dL (↑)	152 mg∕dL (↑)	<100 mg∕dL
	VLDL cholesterol	29 mg∕dL	27 mg∕dL	<30 mg∕dL
	Triglycerides	145 mg∕dL	133 mg∕dL	<175 mg∕dL**
*Protein metabolism*	Total protein	6.7 g∕dL	6.2 g∕dL	6.0–8.3 g∕dL
	Albumin	4.3 g∕dL	4.3 g∕dL	3.5–5.2 g∕dL
	Globulin	2.3 g∕dL	1.9 g∕dL	1.7–3.5 g∕dL
*Muscle metabolism*	Muscle CK	93 U∕L	243 U∕L (↑)	<170 U∕L
*Liver function*	AST	29 U∕L	32 U∕L	10–40 U∕L
	ALT	26 U∕L	24 U∕L	10–40 U∕L
	GGT	31 U∕L	22 U∕L	5–43 U∕L
*Renal function*	Urea	46 mg∕dL	31 mg∕dL	10–50 mg∕dL
	Creatinine	0.79 mg∕dL	1.38 mg∕dL (↑)	0.6–1.2 mg∕dL
*Iron metabolism*	Iron	105 ug∕dL	108 ug∕dL	60–140 ug∕dL
	Ferritin	23 ug∕dL (↓)	185 ug∕dL	30–300 ug∕dL
	TIBC	301 ug∕dL	298 ug∕dL	250–450 ug∕dL
	TF_sat_	35%	36%	25%–50%
*Other Minerals*	Calcium	8.8 mg∕dL	10.4 mg∕dL	8.6–10.3 mg∕dL
	Zinc	133 ug∕L (↑)	62 ug∕L (↓)	70–120 ug∕L

* Reference value for adult women ** Reference value for non-fasting blood collections.

CK, creatine kinase; AST, aspartate aminotransaminase; ALT, alanine aminotransferase; GGT, gamma-glutamyl transpeptidase; TIBC, total iron-binding capacity; TF_sat_, transferrin saturation index.

C-reactive protein (CRP) was also measured in serum and showed a low value of 0.43 mg/dL (reference value <0.50 mg/dL), with no evidence of clinically relevant systemic inflammation at the time of evaluation. Notably, CRP values obtained over the previous 5 years were consistently low, with a mean of 0.26 mg/dL, indicating that this profile is stable over time and further supporting the interpretation of a low inflammatory background in the TS patient.

### 3.3 Endocrine profile

The patient’s endocrine profile was assessed in collaboration with another clinical laboratory, with samples collected in the morning after an overnight fast and a period of rest. The analyses included parameters of glucose metabolism (fasting blood glucose, insulin, and glycated hemoglobin) and thyroid function (thyroid-stimulating hormone [TSH] and free thyroxine [free T4]).

The patient presented a fasting plasma glucose level of 82 mg/dL (reference values: 75–99 mg/dL), glycated hemoglobin of 5.6% (values below 5.7% indicate a low risk of developing diabetes), and a serum insulin level of 2 µU/mL (reference values: 2–13 µU/mL), all of which were within the normal reference ranges. Based on fasting glucose and insulin values, the patient’s HOMA-IR (Homeostasis Model Assessment of Insulin Resistance) was calculated as 0.49 (reference value <2.35), indicating the absence of insulin resistance or metabolic syndrome. The TSH level was 1.68 mIU/L (reference range: 0.40–7.90 mIU/L) and the free T4 level was 1.10 ng/dL (reference range: 0.9–1.7 ng/dL), both falling within the normal reference intervals. These values indicate a euthyroid state under levothyroxine replacement therapy (37.5 µg/day, approximately 0.9 μg/kg), which corresponds to a low to moderate dose. Considering that the full replacement dose is around 1.5 μg/kg/day, this suggests that a portion of the patient’s thyroid function may still be active. However, due to the use of exogenous hormone, it is not possible to precisely determine the extent of endogenous thyroid hormone production or the integrity of the hypothalamic-pituitary-thyroid feedback axis.

## 4 Discussion

This rare case demonstrates that preserved functional status into older age can occur in individuals with Turner syndrome, a condition classically associated with reduced life expectancy and increased cardiometabolic and skeletal risk (Stochholm et al., 2006; Fuchs et al., 2019). Although the patient never received growth hormone (GH) therapy, which is commonly recommended during childhood for TS patients to improve height and body composition (Danowitz and Grimberg, 2022), early diagnosis followed by decades of continuous estrogen replacement may have played a central role in supporting long-term cardiovascular, cognitive, and bone health. Importantly, her strong familial background of longevity, with a centenarian mother (currently alive at age 101) and a father who survived until 87 years of age without fatal cardiovascular events, suggests that inherited factors may have contributed to survival beyond what is typically expected in TS and potentially mitigate disease-associated risks.

Longevity is a complex, polygenic trait influenced by the cumulative effect of favorable alleles. The sum of favorable alleles, which can be interpreted as a genetic lottery that benefits the descendants of centenarians, may function as a protective, polygenic background that delays or allows an individual to avoid major age-related complications, enabling exceptional lifespan. Favorable alleles for exceptional longevity often relate to metabolic efficiency and protection against age-related diseases (e.g., dyslipidemia and cardiovascular disease). It is worth noting that many genetic variants with the potential to promote longevity are still unknown, especially in admixed populations, and that this is beyond the scope of this study. However, the case of a centenarian’s daughter with TS and a lifespan exceeding expectations is intriguing. It suggests that particular genetic variants present in healthy centenarians may be protective even in the presence of a genetic pathogenic condition.

Turner syndrome itself represents a natural model of X-linked gene dosage imbalance (Abramowitz et al., 2014), offering a unique human framework to explore how reduced X chromosome dosage interacts with endocrine modulation, genetic background, and aging-related pathways. The genetic profile identified in this patient provides additional insight into potential mechanisms underlying her preserved functionality into older age. The coexistence of homozygous variants in genes related to cellular protection (*KL*), lipid metabolism (*CETP*), and inflammatory regulation (*LTA*/*TNF*), together with variants in pathways involved in lipid transport (*APOE*, *APOC1*, *LPL*, *LPA*) and immune modulation (*IL6*), highlights the convergence of biological processes potentially implicated in healthy aging and longevity (Glatt et al., 2007), and reinforces that inherited genetic background may be favoring her biological resilience and longevity.

For instance, the *KL*-VS haplotype has been consistently associated, in meta-analyses, with exceptional longevity, albeit with modest effect sizes, and has been implicated in mechanisms related to cardiovascular protection, oxidative stress regulation, insulin signaling, and maintenance of metabolic homeostasis (Revelas et al., 2018). Enrichment of the *KL*-VS haplotype among long-lived individuals supports a contributory role of Klotho in resilience to age-related physiological decline. Furthermore, the concordance between genotype and phenotype supports a functional contribution of the *CETP* rs7499892 variant to a protective lipid profile, while acknowledging the multifactorial regulation of lipid metabolism. The *CETP* rs7499892 C/C genotype has been associated with higher circulating HDL cholesterol levels in large population-based and heritability studies (van Leeuwen et al., 2015). Moreover, other variants identified in the TS patient (e.g., *APOC1* rs4420638 A/A, *LPL* rs268 A/A, *PABPC4* rs3768321 G/G) have also been associated with elevated HDL cholesterol levels in population-based studies.

The TS patient is also homozygous for the *TNF* variant rs1800629 G-allele, located in the promoter region of the *TNF* gene and associated with reduced *TNF-α* expression (Bi et al., 2012), which is in accordance with laboratory findings of our TS patient. This genotype has been reported at higher frequencies among individuals with tightly regulated inflammatory responses and favorable immune profiles, including long-lived populations. Although each variant confers only a small to modest effect, their combined presence may contribute to a biological milieu characterized by enhanced cellular homeostasis, favorable lipid handling and attenuated chronic inflammation, consistent with her observed clinical and biochemical phenotype.

Rather than reflecting isolated laboratory findings, the TS patient’s hematological and biochemical profiles collectively point to preserved physiological regulation for her age. Stable blood counts, low erythrocyte size variability, and the absence of clinically significant anemia suggest maintained hematopoietic function, while a favorable cardiometabolic balance—characterized by high HDL cholesterol, normal triglycerides, and preserved hepatic, renal, and muscular markers—indicates sustained systemic homeostasis. Together, these features support the concept of metabolic and inflammatory resilience, despite the presence of lifelong monosomy X and age-related comorbidities.

While cardiovascular complications are a major determinant of mortality in Turner syndrome, polygenic risk score (PRS) analyses for cardiovascular traits (such as aortic dilation and hypertension) were beyond the scope of this case report. Notably, despite the expected elevated cardiovascular risk, the patient did not present aortic dilation or major structural abnormalities on echocardiographic evaluation, consistent with a potentially protective phenotype. Future studies in larger cohorts will be necessary to determine whether protective or deleterious polygenic profiles contribute to cardiovascular risk and resilience in this population.

## 5 Conclusion

Overall, this case suggests that a favorable genetic background together with an early diagnosis and sustained hormone replacement therapy may promote resilience and healthy aging despite disease-associated risks. Although causality cannot be inferred from a single case, the convergence of genetic and adequate management support a biologically coherent framework contributing to this atypical healthspan in Turner syndrome.

## Data Availability

The data supporting the findings of this case report are available from the corresponding author upon reasonable request. The data are not publicly available due to privacy and ethical considerations.

## Supplementary material

SUPPLEMENTARY FIGURE S1 Transthoracic echocardiographic assessment. 2D images demonstrating preserved left ventricular systolic function, with an estimated ejection fraction of ∼60% (Teichholz method). Left ventricular dimensions, wall thickness, and derived indices are within normal reference ranges. The aortic root diameter is normal, with no evidence of dilation.
