# Supplementary figures and images for: Case Report: Exceptional longevity in turner syndrome
Source: Front Aging. 2026 Jun 10;7:1805342. doi: 10.3389/fragi.2026.1805342 (PMC13291543; doi:10.3389/fragi.2026.1805342)

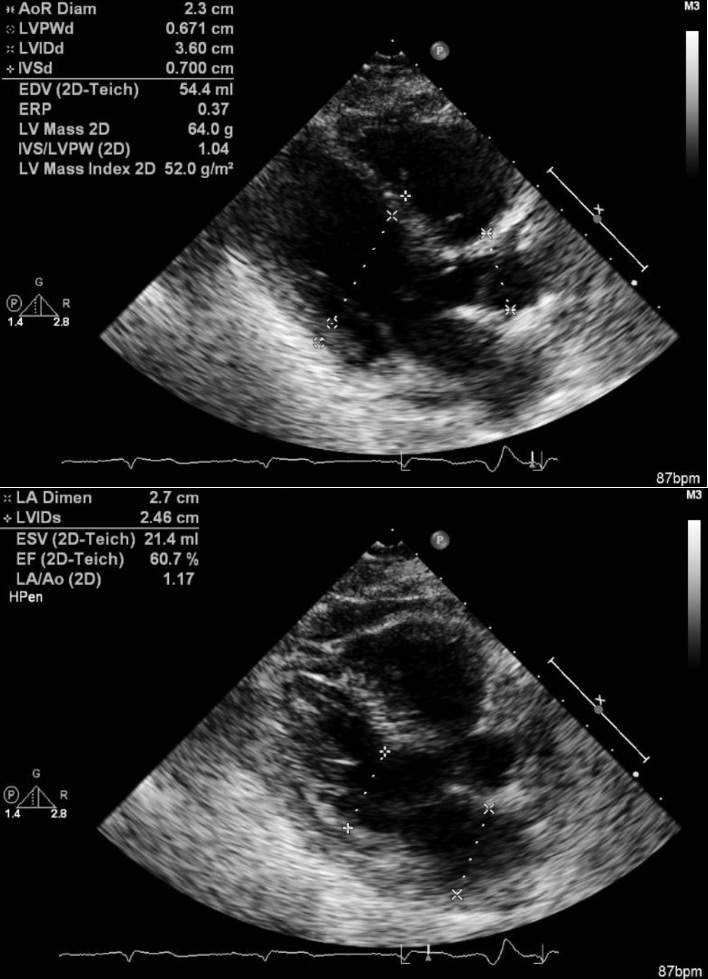

Supplement: Supplementary file 1 [file Image1.jpeg]
